# Characterization and comparative genomic analysis of a marine *Bacillus* phage reveal a novel viral genus

**DOI:** 10.1128/spectrum.00037-24

**Published:** 2024-08-20

**Authors:** Min Jin, Meishun Yu, Xuejin Feng, Yinfang Li, Menghui Zhang

**Affiliations:** ^1^State Key Laboratory Breeding Base of Marine Genetic Resource and Southern Marine Science and Engineering Guangdong Laboratory (Zhuhai), Third Institute of Oceanography, Ministry of Natural Resources, Xiamen, China; 2 College of Ocean and Earth Sciences, Xiamen University, Xiamen, China; Centre de Biologie Integrative, Toulouse, France

**Keywords:** *Bacillus *phage, *Bacillus pumilus*, myophages, novel genus

## Abstract

*Bacillus pumilus* exhibits substantial economic significance, with its metabolism, adaptability, and ecological functions regulated by its bacteriophages. Here, we isolated and characterized a novel temperate phage vB_BpuM-ZY1 from *B. pumilus* derived from mangrove sediments by mitomycin C induction. Phage vB_BpuM-ZY1 is a typical myophage, which has an icosahedral head with a diameter of 43.34 ± 2.14 nm and a long contractible tail with a length of 238.58 ± 5.18 nm. Genomic analysis indicated that vB_BpuM-ZY1 encodes genes for lysogeny control, and its life cycle may be intricately regulated by multiple mechanisms. vB_BpuM-ZY1 was predicted to employ P2-like 5'-extended-cos packaging strategy. In addition, genome-wide phylogenetic tree and proteome tree analyses indicated that vB_BpuM-ZY1 belongs to the *Peduoviridae* family but forms a separate branch at a deeper taxonomic level. Particularly, the comparative genomic analysis showed that vB_BpuM-ZY1 has less than 70% intergenomic similarities with its most similar phages. Thus, we propose that vB_BpuM-ZY1 is a novel *Bacillus* phage belonging to a new genus under the *Peduoviridae* family. The protein-sharing network analysis identified 44 vB_BpuM-ZY1-related phages. Interestingly, these evolutionarily related myophages infect a broad range of hosts across different phyla, which may be explained by the high structural variations of the host recognition domain in their central spike proteins. Collectively, our study will contribute to our understanding of *Bacillus* phage diversity and *Bacillus*-phage interactions, as well as provide essential knowledge for the industrial application of *B. pumilus*.

**IMPORTANCE:**

Although recent metagenomics research has obtained a wealth of phage genetic information, much of it is considered “dark matter” because of the lack of similarity with known sequences in the database. Therefore, the isolation and characterization of novel phages will help to interpret the vast unknown viral metagenome data and improve our understanding of phage diversity and phage-host interactions. *Bacillus pumilus* shows high economic relevance due to its wide applications in biotechnology, industry, biopharma, and environmental sectors. Since phages influence the abundance, metabolism, evolution, fitness, and ecological functions of bacteria through complex interactions, the significance of isolation and characterization of novel phages infecting *B. pumilus* is apparent. In this study, we isolated and characterized a *B. pumilus* phage belonging to a novel viral genus, which provides essential knowledge for phage biology as well as the industrial application of *B. pumilus*.

## INTRODUCTION

Viruses are the most abundant biological entities in the ocean, with approximately ten times the abundance of prokaryotes (1). The majority of marine viruses are phages that infect bacteria. It is estimated that phage infections cause an estimated 20%–40% of prokaryotic mortality per day in the ocean (2). Phages possess high morphological and genetic diversity, regulate the community structure, metabolism, fitness, and evolution of hosts, and have enormous implications in marine biogeochemical cycles (3, 4). During infection, phages can execute a lytic strategy to synthesize viral particles immediately after infection and release them by lysing hosts or a lysogenic strategy to integrate into and replicate with the host genome until being induced into the lytic cycle (5). Marine phages have enormous undescribed genetic diversity (6, 7). Although recent metagenomics researchers have obtained a wealth of phage genetic information, much of it is considered “dark matter” because of the lack of similarity with known sequences in the database (8). Besides, metagenomic data cannot provide in-depth information on interactions between a specific phage-host pair. It has been suggested that these problems could be partially solved by the isolation and characterization of novel culturable phages (8, 9). Therefore, the isolation and characterization of novel marine phages will help to interpret the vast unknown viral metagenome data and improve our understanding of marine phage diversity and phage-host interactions.

*Bacillus* is an endospore-forming, Gram-positive, and rod-shaped bacterial genus with many members inhabiting different ecological niches. Some members of *Bacillus* have been applied in industrial, agricultural, medical, and food-related fields (10–13). For example, *Bacillus licheniformis*, *Bacillus subtilis,* and *Bacillus amyloliqueficiens* have great potential to contribute to the continuity of farming of fish by inhibiting pathogens and promoting fish immunity and other functions (11). The δ-endotoxins (Cry and/or Cyt proteins) produced by *Bacillus thuringiensis* are used as topical pesticides to protect crops (12). However, some members of *Bacillus* are pathogenic to humans, including *Bacillus anthracis*, which is the causative agent of anthrax, and some *Bacillus cereus*, which is commonly known to cause food poisoning (14). *Bacillus pumilus* is commonly found in a variety of environments, including seawater, deep-sea sediments, and soil, and exhibits significant resistance to environmental stresses such as low nutrient availability, UV radiation, and oxidizing enzymes (15, 16). In addition, *B. pumilus* is considered a plant growth-promoting bacteria with direct or indirect effects on plant growth, such as the production of auxins and antifungal active antibiotics (16). Moreover, *B. pumilus* shows high economic relevance due to its wide applications in biotechnology, industry, biopharma, and environmental sectors, such as being an antimicrobial agent and probiotic for animals and humans (17). Since phages influence the abundance, metabolism, evolution, fitness, and ecological functions of bacteria through complex interactions (4), the isolation and characterization of novel phages infecting *B. pumilus* is of great significance both for industry and phage biology.

As of May 2023, there are 130 linear or circular complete genomes of *Bacillus*-infecting phages in the Viral RefSeq database (Release 218) with sizes of 14,319–251,042 bp. These *Bacillus* phages have different morphological, genomic, and life cycle characteristics (10, 18). However, it is suggested that the reported *Bacillus* phages represent only a small fraction of the existing diversity, and each *Bacillus* genome contained five predicted prophages on average (10, 19). Because temperate phages play important roles in host fitness and evolution, such as the formation of ecologically important and/or industrially relevant bacterial traits (e.g., virulence factors), the isolation and characterization of temperate phages inhabiting the *Bacillus* genome is essential for the industrial application of *Bacillus* strains. In this study, we induced a novel temperate phage (vB_BpuM-ZY1) from *B. pumilus* by mitomycin C and characterized its morphological, physiological, and genomic features. This phage represents a new genus of *Bacillus* phage with low similarity with known phages.

## RESULTS

### Morphology of phage vB_BpuM-ZY1

**Fig 1:**
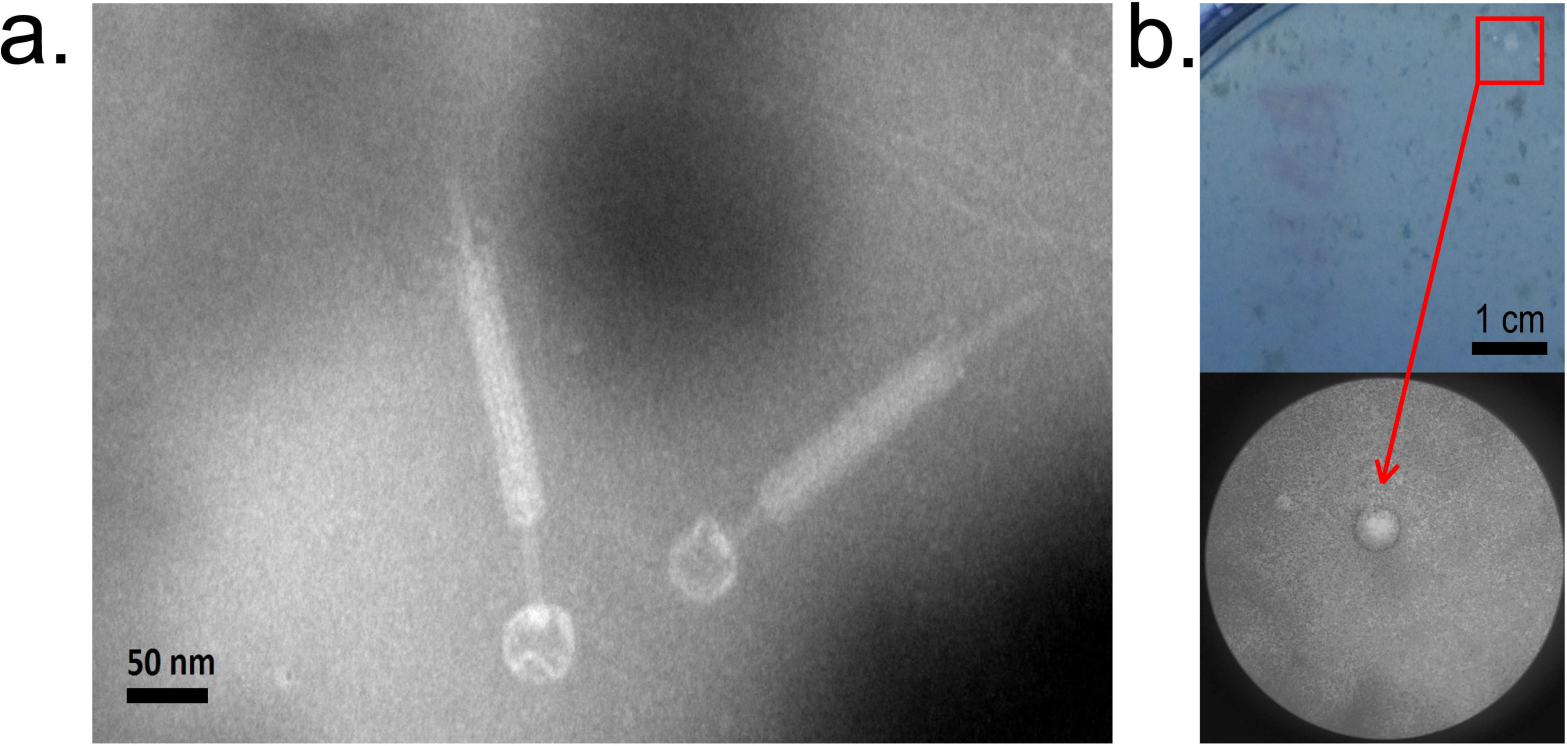
Morphology of phage vB_BpuM-ZY1. (a) TEM of vB_BpuM-ZY1. Phage particles were stained with 1% (wt/vol) phosphotungstic acid and examined with TEM at 80 kV. (b) Plaque morphology of vB_BpuM-ZY1 on double-layer agar plates.

A novel *Bacillus* phage vB_BpuM-ZY1 was induced from *B. pumilus* derived from the mangrove sediment in Fujian, China, by mitomycin C. Morphological characterization of vB_BpuM-ZY1 by transmission electron microscopy (TEM) revealed that it is a typical myophage, which has an icosahedral head with a diameter of 43.34 ± 2.14 nm and a long contractible tail with a length of 238.58 ± 5.18 nm (Fig. 1a). Phage vB_BpuM-ZY1 formed round turbid plaques with a diameter of approximately 1.50 mm on double-layer agar plates (Fig. 1b).

### Genomic analysis of vB_BpuM-ZY1

**Fig 2:**
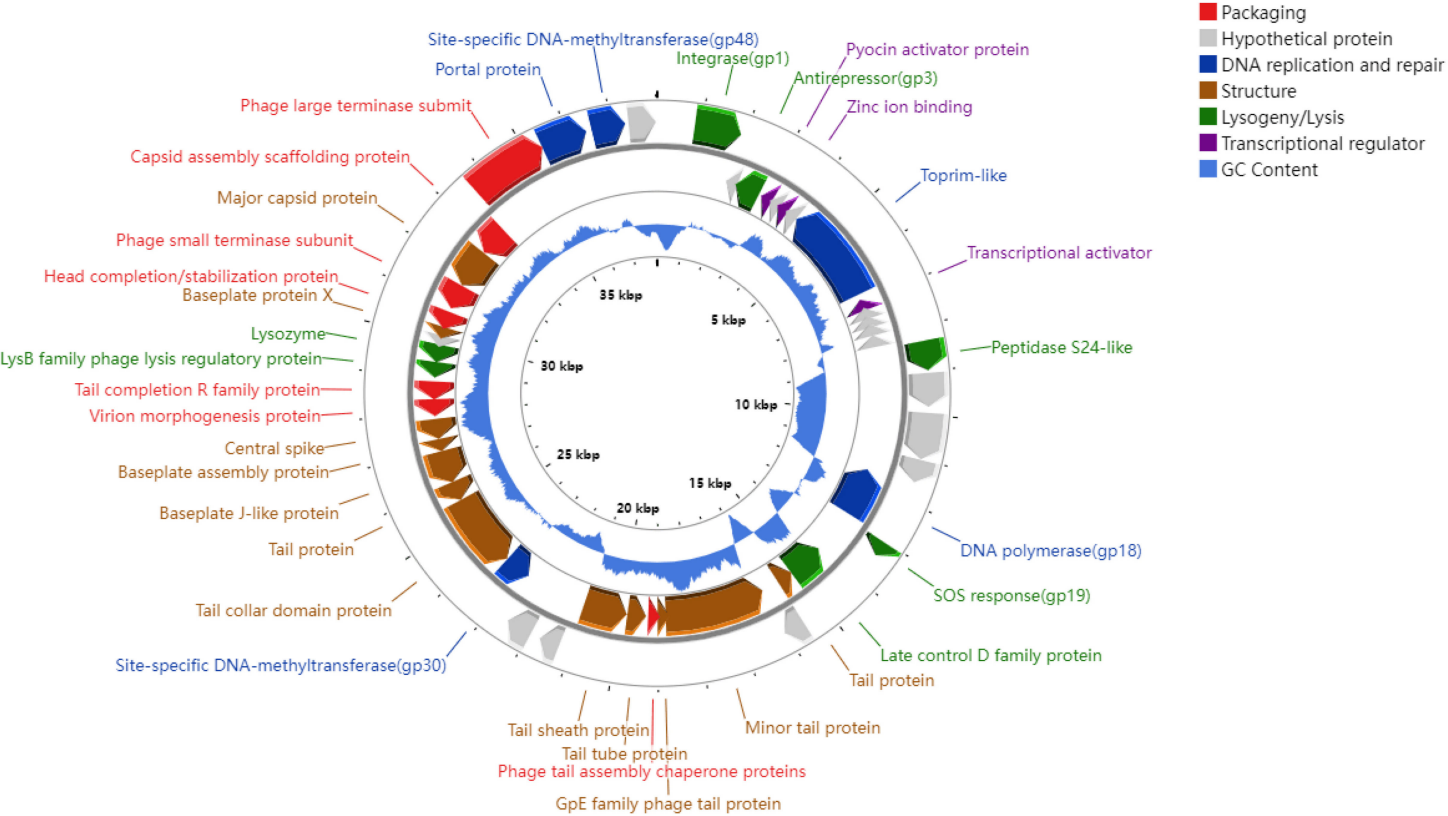
Genomic map of vB_BpuM-ZY1. From inner to outer rings: scale bar for genome position, GC content, genes transcribed counterclockwise, and genes transcribed clockwise. Genes are colored according to their annotated functional modules.

vB_BpuM-ZY1 has a double-stranded DNA genome with a length of 37,161 bp and a GC content of 58.4%. The genome was predicted to encode 49 genes, ranging from a minimum sequence length of 137 bp to a maximum of 2,666 bp. Among these, 15 genes were identified as putative proteins, while 34 genes were functionally annotated and categorized into various modules, including DNA packaging, DNA replication and repair, structure, lysogeny and lysis control, and transcriptional regulation (Table S1). The genomic map of vB_BpuM-ZY1 is depicted in Fig. 2, with the integrase located at the beginning and the transcriptional direction of most genes counterclockwise. The presence of tRNA in phages may be related to different codon and amino acid usage from the host (20). However, tRNA-encoding genes were not found in the vB_BpuM-ZY1 genome, suggesting that vB_BpuM-ZY1 may have a tight and specific relationship with *B. pumilus* (20).

Approximately 20% of isolated phage genomes contain methyltransferase-like genes that help phages evade the hosts’ restriction-modification system, thereby increasing their ability to infect the hosts (21). We identified two methyltransferase-encoding genes (gp30 and gp48) in the genome of vB_BpuM-ZY1, which may improve its success in infecting hosts. In addition, methyltransferases may be of specific significance to lysogeny. A model of phage life-cycle regulation suggests that adenine methyltransferase methylates the antirepressor gene (22). Once methylation is removed, the repressor is inhibited, and the phage exits the lysogenic state, entering the lytic cycle (22). The simultaneous occurrence of methyltransferase genes (gp30 and gp48) and antirepressor gene (gp3) in vB_BpuM-ZY1 genome suggests that the life cycle of vB_BpuM-ZY1 may also be regulated by methyltransferase. Interestingly, we also identified an SOS response protein (gp19) in the genome. Some factors inducing the transition of temperate phages from lysogenic to lytic cycles are considered to be coupled with the host SOS response (4). Taken together, as a temperate phage, the life cycle of vB_BpuM-ZY1 may be intricately regulated by multiple mechanisms.

### Protein-sharing network of vB_BpuM-ZY1 with known phages

**Fig 3:**
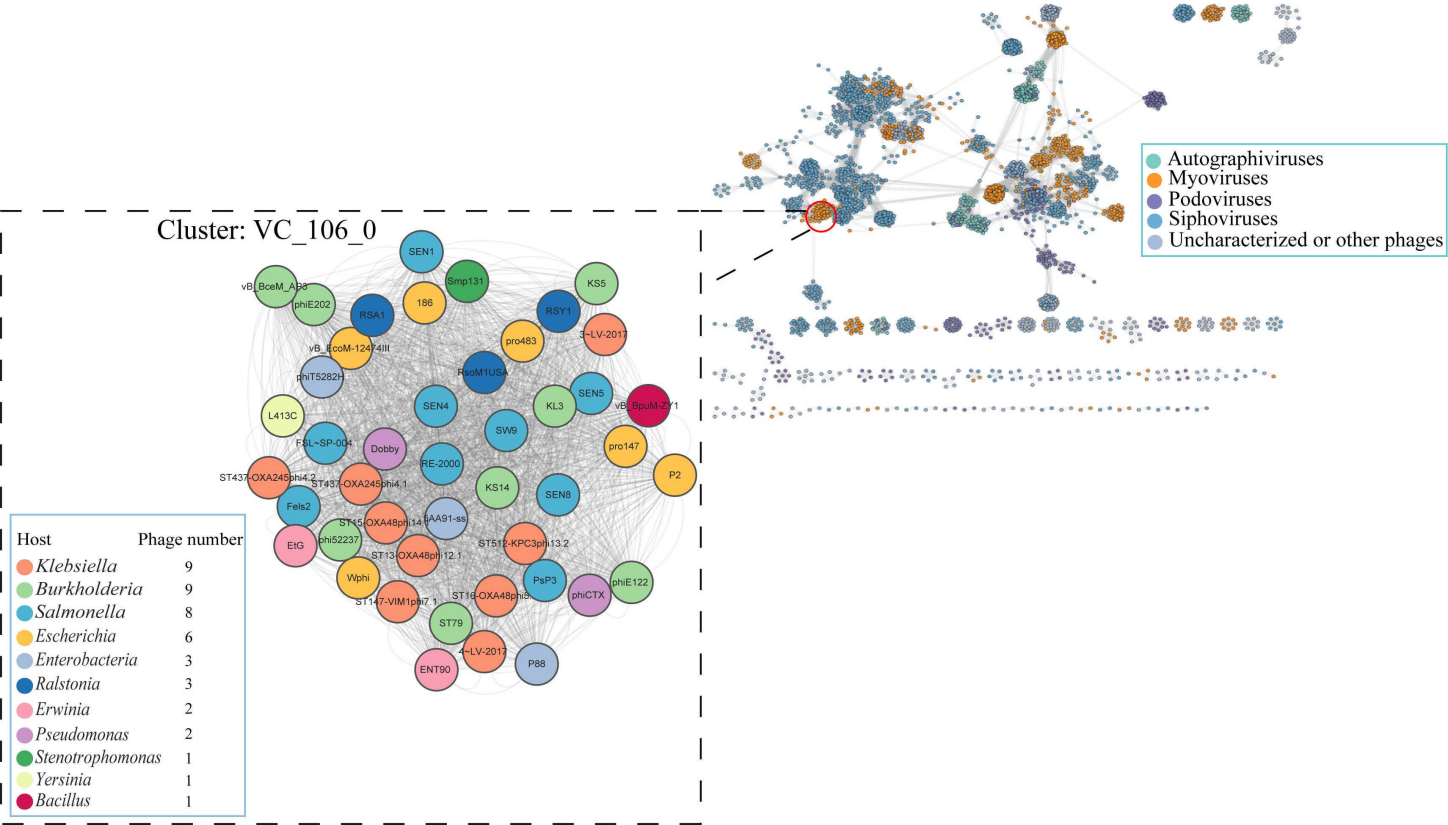
Protein-sharing network of vB_BpuM-ZY1 and 3503 known Phages generated with vConTACT2. Phages (nodes) are connected by edges, indicating the significant pairwise similarity between them in terms of shared protein contents. The subnet (VC_106_0) of vB_BpuM-ZY1 and its related phages are enlarged inside.

Based on the viral clusters (VCs) formed by shared proteins, the protein-sharing network was generated on the genomes of vB_BpuM-ZY1 and 3503 known phages to analyze the location of vB_BpuM-ZY1 in the phage universe (Fig. 3; Table S2). The results showed that vB_BpuM-ZY1 was located in the same cluster (VC_106) as 44 myophages, including Pseudomonas phage phiCTX (NC_003278.1, isolated from bovine mastitis) (23), which had similar genomic features with vB_BpuM-ZY1 including genome size and GC content. Notably, these evolutionarily related myophages infect multiple Gram-positive and negative hosts across the different phylum, showing a broad host range.

### vB_BpuM-ZY1 belongs to a novel genus under the *Peduoviridae* family

**Fig 4:**
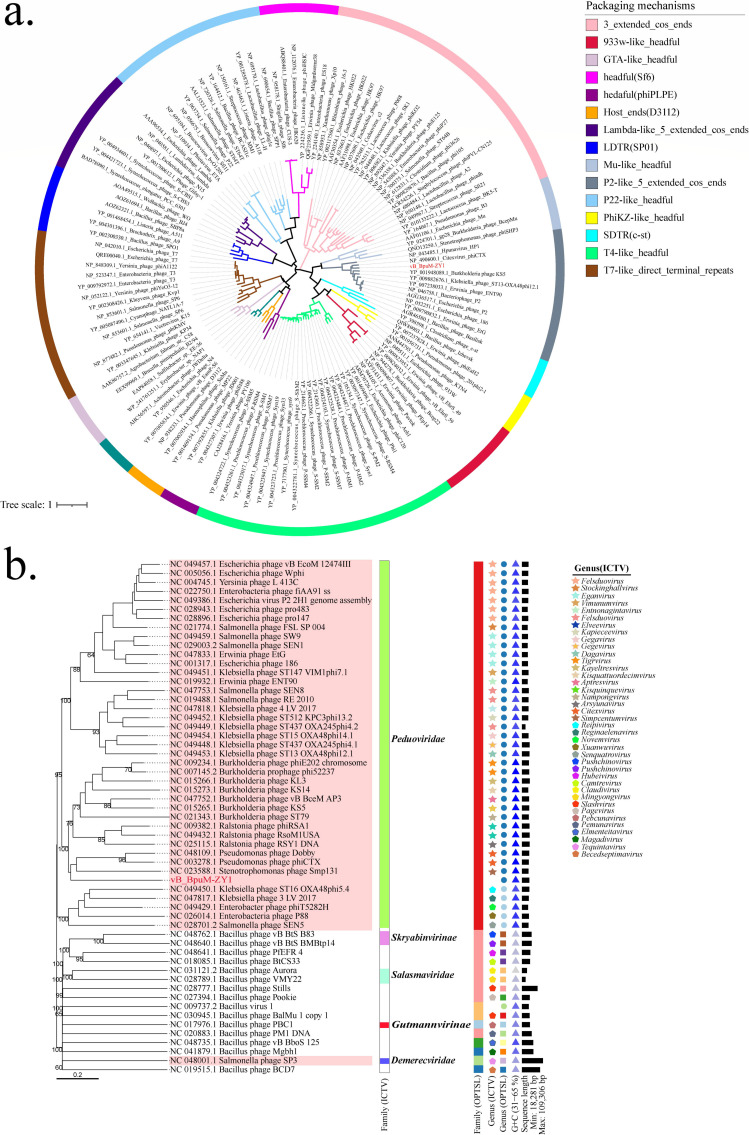
Phylogenetic analysis of vB_BpuM-ZY1. (a) Large terminase subunit (TerL)-based tree of vB_BpuM-ZY1 and 127 other phages with known packaging mechanisms. The tree was generated by iqtree using the maximum-likelihood method with a bootstrap value of 1,000. The branches are colored according to the types of packaging mechanisms. The position of vB_BpuM-ZY1 in the tree is indicated with red color. (b) The genome BLAST distance phylogeny tree of 57 phage genomes was reconstructed by Virus Classification and Tree Building Online Resource (VICTOR) with the D6 formula and 56% average support. The different shapes and colors represent the clusters of ICTV and OPTSIL at the family- and genus-levels. Phages from the cluster of VC_106 generated by protein-sharing network analysis (Fig. 3) are shaded in pink.

To predict the packaging mechanism and identify the genetic relationships of vB_BpuM-ZY1, we constructed a phylogenetic tree based on the large terminase subunit (TerL) of vB_BpuM-ZY1 and 127 other phages with known DNA packaging mechanisms (24). TerL is responsible for the ATP-powered translocation of phage genomic DNA into the phage procapsids at the late phase of infection and serves as a good marker for taxonomic classification and packaging strategies prediction in *Caudoviricetes* (10). As shown in Fig. 4a, vB_BpuM-ZY1 fell into the same branch as Pseudomonas virus phiCTX, Burkholderia phage KS5, and Escherichia phage P2, among other phages known to use the P2-like 5'-extended-cos packaging mechanism, suggesting that these phages are close relatives. In addition, we used the genomes of vB_BpuM-ZY1 and 56 related phages (41 VC_106 phages and an additional 15 *Bacillus* phages) to reconstruct the genome BLAST distance phylogeny (GBDP) tree (Fig. 4b). The OPTSIL clustering yielded 57 species-level clusters, 15 genus-level clusters, and seven family-level clusters. Except for Salmonella phage SP3, all VC_106 phages fell into the same family-level cluster, and this result was also consistent with their classification in the International Committee on Taxonomy of Viruses (ICTV) as *Peduoviridae*. At the genus level, most of the members of VC_106 phages fell into the same genus-level cluster according to OPTSIL. However, they were classified into 20 different genera by ICTV. Consistently with the GBDP tree, the proteome tree also showed that vB_BpuM-ZY1 clustered with Stenotrophomonas phage Smp131, Pseudomonas phage phiCTX, and Pseudomonas phage Dobby (NC_048109.1, isolated from kidney stone) but in different branches (Fig. S1) (25). Similar to the phylogenetic analysis of TerL, the GBDP tree and proteomic tree indicated that vB_BpuM-ZY1 was most similar to *Pseudomonas*, *Stenotrophomonas*, *Ralstonia,* and *Burkholderia* phages but showed lower correlation with *Bacillus* phages, suggested that vB_BpuM-ZY1 represents a novel *Bacillus* phage that could be grouped into *Peduoviridae* family.

**Fig 5:**
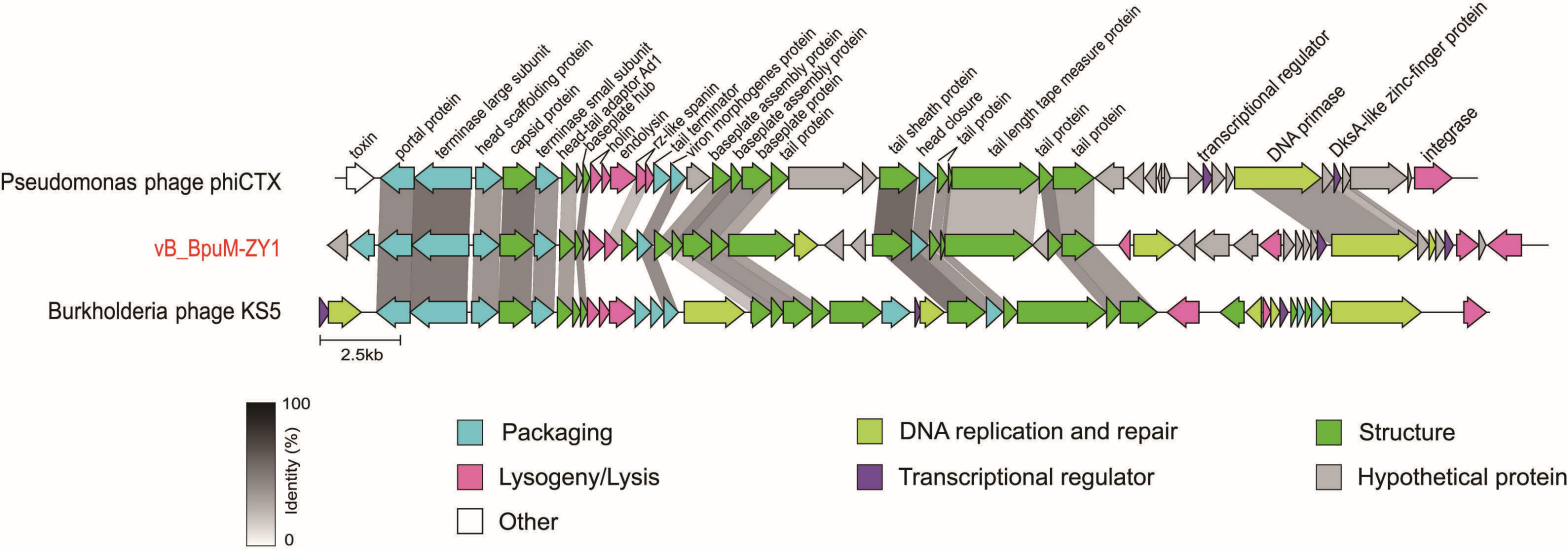
Genome comparison of vB_BpuM-ZY1 with its close relatives. Genes are colored according to their annotated functional modules. Arrows represent the direction of gene transcription, and the gradient scale indicates the sequence identity range between phage genes.

The comparative genomic analysis showed isomorphic modules and gene synteny similar between vB_BpuM-ZY1, Pseudomonas phage phiCTX, and Burkholderia phage KS5 (NC_015265.1) (26), with identity values more than 25% (Fig. 5). Most of the homologous regions in these three genomes were located in the left and middle arms, encompassing genes encoding structural, and packaging proteins. These genes are functionally important for the morphogenesis of phage particles, indicating an evolutionary conservation.

**Fig 6:**
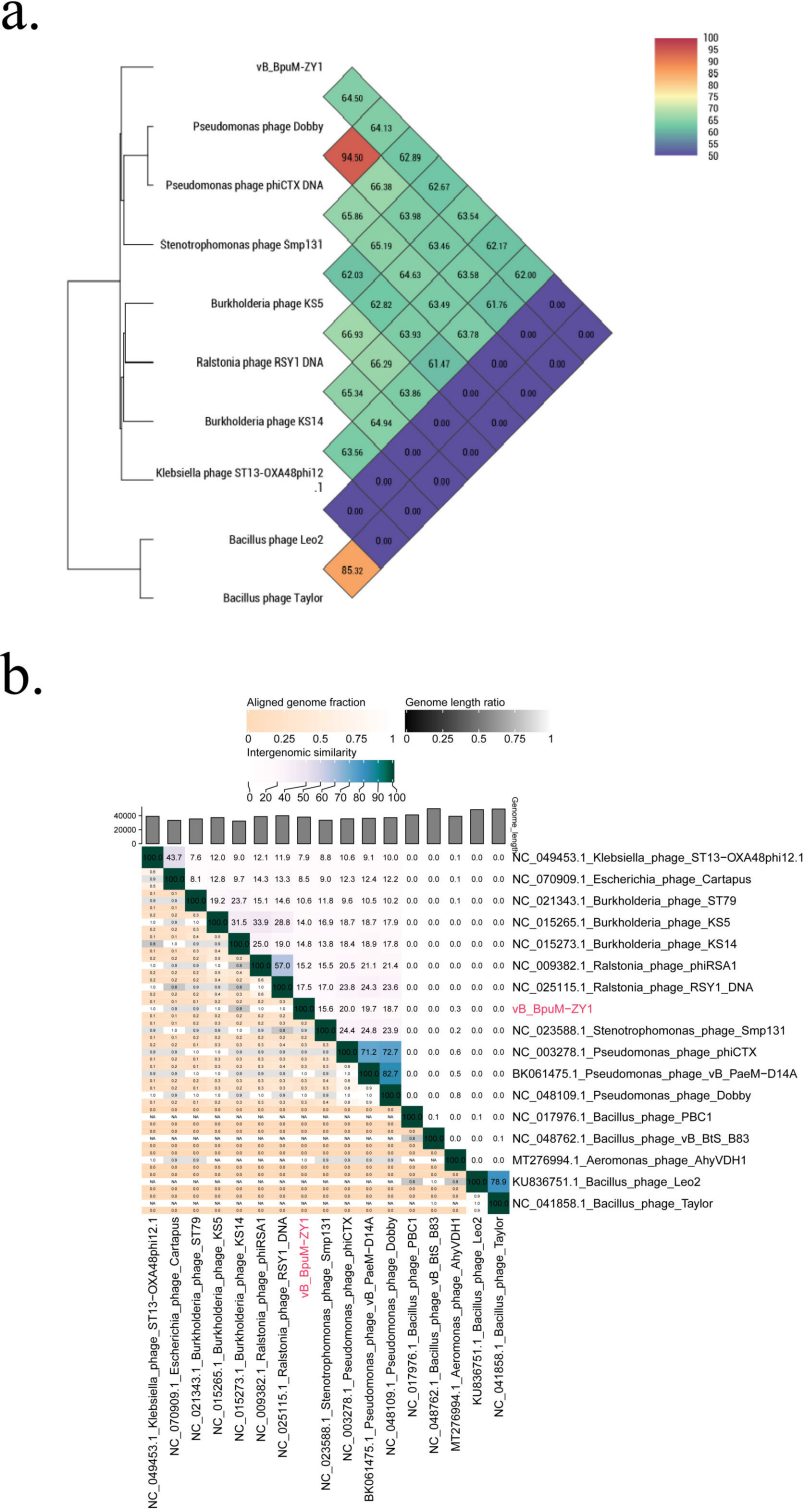
Intergenomic similarities between vB_BpuM-ZY1 and its related phages. (a) The average nucleotide identity values between pairwise phage genomes were calculated by OrthANI software. (b) The intergenomic similarity between vB_BpuM-ZY1 and its related phages was generated using VIRIDIC. The right half shows the similarity values between phage genomes. The left half indicates the aligned genome fraction and genome length ratio for a selected pair of phage genomes.

According to ICTV’s demarcation criteria for tailed phages (*Caudoviricetes* class), when the intergenomic similarity between phages is greater than 70%, they are classified as the same genus, and when it exceeds 95%, they are considered as the same species (27). As shown in Fig. 6a, the average nucleotide identity (ANI) values between vB_BpuM-ZY1 and its close relatives were 62.00%–64.50%. Since the ANI tools may normalize the intergenomic identities to the length of the alignment rather than the whole genome length, leading to artificially high genomic similarities (28), we further analyzed the similarities between vB_BpuM-ZY1 and its close phages using the whole genome length-based algorithm of VIRIDIC (Fig. 6b). The heatmap showed that intergenomic similarities between vB_BpuM-ZY1 and these close phages range from 0 to 20%, with phiCTX 20%, vB_PaeM−D14A 19.7% (BK061475.1, isolated from Portuguese and Spanish critically ill patients), and Dobby 18.7% being the highest (25, 29). These findings are consistent with the results obtained from ANI and phylogenetic analyses. Collectively, these results suggest that vB_BpuM-ZY1 belongs to a novel genus under the *Peduoviridae* family.

### Sequence analysis and 3D structure of vB_BpuM-ZY1 central spike

Recognition and adsorption of the phage to the host is the initiation point of infection. In myophages, such as Escherichia phage P2, this process is mediated by phage tail fibers and central spike (30). The central spike is one of the key proteins of the contractile injection system in myophages (31). It creates an opening in the host’s cell membrane during infection, which is a prerequisite for the subsequent entry of phage genomic DNA into the host cell (32, 33). The results of the above protein-sharing network analysis showed that members of the evolutionarily related myophages in VC_106 infect a broad range of hosts across phylum (Fig. 3). To find the reasons for this unusual phenomenon, we compared the amino acid sequences and 3D structure of central spikes among vB_BpuM-ZY1 and its closest relatives in VC_106. vB_BpuM-ZY1 gp35 (ZY1-gp35), contains 183 amino acids, was annotated as a central spike. Sequence analysis showed that ZY1-gp35 is most similar to the central spike of phage P2 (P2-gpV), another member in the VC_106 cluster, based on the predictions of HHpred (probability: 99.93%, E-value: 8.7e-23, score: 146.36, aligned cols: 180, identities: 39%) and Phyre2 (confidence: 100%, coverage: 85%, identity: 34%).

**Fig 7:**
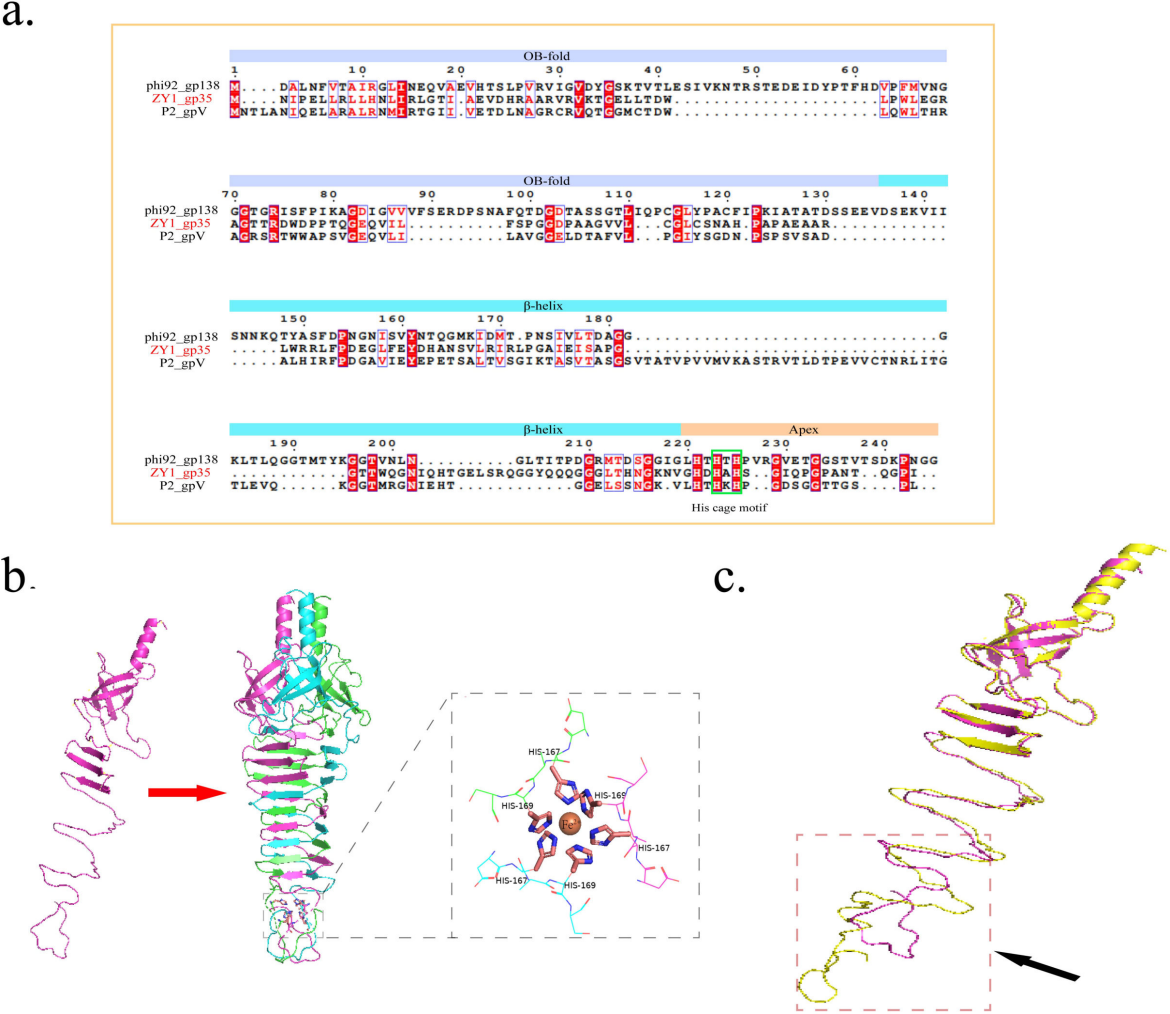
Sequence analysis and 3D structure of vB_BpuM-ZY1 central spike protein. (a) Multiple alignments of the amino acid sequences of central spike proteins of vB_BpuM-ZY1 (ZY1-gp35), phage P2 (P2-gpV), and phage phi92 (phi92-gp138). The identical residues through all aligned sequences are colored in red, and the histidine residuals H167 and H169 that form the histidine cage were marked with green boxes. (b) The 3D structure of the monomer (left) and trimer (right) of ZY1-gp35. Different subunits are represented by different colors. At the C-terminus, the histidine residual H167 and H169 at each subunit form an octahedral intermolecular histidine cage with an iron ion buried in its center. The 3D structures of the monomer and trimer of ZY1-gp35 were predicted by Alphafold2. (c) Superposition of the 3D model of ZY1-gp35 (violet) with the crystal structure of P2-gpV (yellow). The structural variations are indicated with black arrows. The 3D structure of the monomer ZY1-gp35 was predicted by Alphafold2.

The multiple alignments of the amino acid sequences of ZY1-gp35 with P2-gpV and phi92-gp138 (central spike of Escherichia phage phi92 that is homologous to P2-gpV) are shown in Fig. 7a. Three conserved domains were identified in ZY1-gp35, including an N-terminal OB-fold domain that is responsible for attaching to the phage baseplate, a β-Helix domain, and an Apex domain (32, 33). The HxH motif (histidine 167-alanine 168-histidine 169) was further identified in the Apex domain of ZY1-gp35, which is the only conserved motif of the central spike in myophages with contractile tail (32, 34). A generally conserved feature of the central spike trimer is that the histidine residues in the Apex domain form an octahedral intermolecular histidine cage with an iron ion buried in its center, which is also observed in the 3D structure of the trimer of ZY1-gp35 (Fig. 7b) (32, 33, 35).

3D structure modeling using P2-gpV as the template indicated that the 3D structures of ZY1-gp35 and P2-gpV showed high similarity at the N terminus. However, there were significant structural variations at the C terminus (Fig. 7c). Previous studies have shown that the C-terminal domain is necessary and sufficient for the binding of P2-gpV to the cell membrane of *Escherichia coli* (30, 36). Therefore, structural differences in the C terminus of the central spike may explain why vB_BpuM-ZY1 infects different hosts from its closest relatives. Interestingly, in phage P2, the first step of host adsorption is reversibly achieved by its tail fibers before the central spike protein interacts with the cell membrane (30, 37). However, we did not identify the gene encoding for tail fibers in the genome of vB_BpuM-ZY1 (Table S1), and TEM observation did not reveal a structure similar to tail fiber (Fig. 1a), suggesting that vB_BpuM-ZY1 may use a different host recognition mechanism from phage P2.

### Sequence analysis and 3D structure of vB_BpuM-ZY1 endolysin

**Fig 8:**
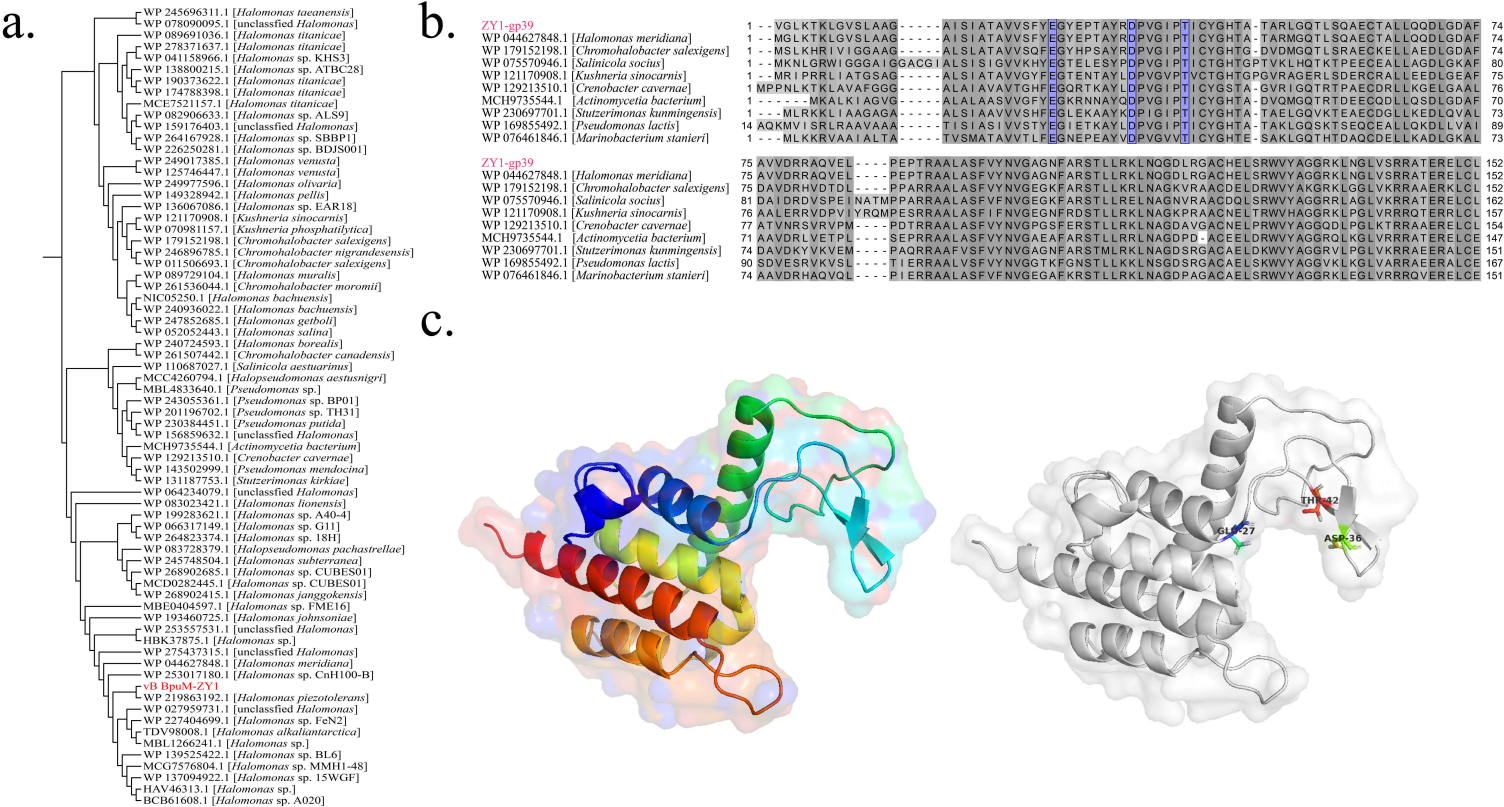
Sequence analysis and 3D structure of vB_BpuM-ZY1 endolysin. (a) Maximum-likelihood phylogenetic tree of vB_BpuM-ZY1 endolysin and the related lysozyme according to protein BLASTp top hit. The position of vB_BpuM-ZY1 in the tree is indicated with red color. (b) Multiple alignments of the amino acid sequences of vB_BpuM-ZY1 endolysin with the closely related lysozyme. The conserved catalytic residues are in blue. (c) 3D structure of vB_BpuM-ZY1 endolysin predicted by Alphafold2. The positions of conserved catalytic residues are indicated.

Phage-encoded endolysin disrupts peptidoglycan in the host cell wall, thereby mediating the release of viral progenies during lysis (38, 39). We identified an endolysin gene from the vB_BpuM-ZY1 genome (ZY1-gp39). Phylogenetic analysis indicated that ZY1-gp39 clustered with the lysozyme of *Halomonas meridiana* (WP_044627848) (Fig. 8a). Conserved domain analysis revealed that these lysozyme belong to the endolysin R21-like proteins (cd16900, a member of the lysozyme-like superfamily), which degrade peptidoglycan in the bacterial cell wall by cleaving glycosidic beta 1,4-bonds between the N-acetylmuramic acid and the N-acetylglucosamine (40). According to the catalytic activity (in terms of the bond they cleave within peptidoglycan), lysozymes are classified into five categories (41), and ZY1-gp39 is predicted to belong to N-acetylmuramidases. Multiple alignments identified three residues in ZY1-gp39 as the conserved catalytic motif (Fig. 8b). Moreover, NCBI CD-Search showed that the receptor binding pocket residues of ZY1-gp39 consisted of glutamate 27, aspartate 36, and threonine 42 (Fig. 8b and c).

## DISCUSSION

In this study, we isolated a novel phage vB_BpuM-ZY1 from *B. pumilus* using mitomycin C induction and conducted a further comprehensive investigation into the biological and genomic characteristics of vB_BpuM-ZY1. vB_BpuM-ZY1 encodes an integrase gene, suggesting that it can follow a temperate phage strategy. The fact that vB_BpuM-ZY1 can be induced by mitomycin C and forms turbid plaques on double-layer agar plates further supports that vB_BpuM-ZY1 is a temperate phage. Interestingly, the lysogenic cycle of vB_BpuM-ZY1 may be intricately regulated by multiple mechanisms, including methylation and host SOS response processes. This result reveals the complex and tight interactions between vB_BpuM-ZY1 and the host. Given that the life cycle transitions of temperate phages potentially cause substantial impacts on the host-phage interactions and relevant biogeochemical cycling (4), different life strategies of vB_BpuM-ZY1 may profoundly influence metabolism and fitness of *B. pumilus* as well as the microbial process mediated by *B. pumilus*. In addition, knowing how different life strategies of vB_BpuM-ZY1 affect the growth and metabolic capabilities of *B. pumilus* during large-scale culture may also be crucial for its industrial production.

Genome-based approaches have now replaced simple morphology-based approaches for phage taxonomic classification (27). In this study, we explored vB_BpuM-ZY1’s genetic relationship with known phages in detail. According to the ICTV demarcation criteria (27), our results revealed that vB_BpuM-ZY1 represents a new phage genus. We therefore propose a new genus-level taxonomy belonging to the *Peduoviridae* family for the new phylogenetic branch represented by vB_BpuM-ZY1. Furthermore, it is noteworthy that vB_BpuM-ZY1 isolated from *B. pumilus* exhibited low genomic correlation with other *Bacillus* phages, whether based on the results of TerL phylogenetic analysis, protein-sharing network analysis, ANI calculation, or comparisons based on genome-wide similarity. Considering the high untapped diversity of *Bacillus* phages, vB_BpuM-ZY1 may exhibit genetic characteristics of a viral lineage that is evolutionarily distant from other known *Bacillus* phages, which will help to reveal the evolutionary history of *Bacillus* phages and improve our understanding of the diversity and taxonomy of *Bacillus* phages (10, 19). In addition, the discovery of novel *B. pumilus* phages will also help to interpret the vast unknown viral metagenome data (9).

Recognition and adsorption are important steps for phage infection (30). Similar to other myophages, vB_BpuM-ZY1 may also be dependent on the central spike to pierce the host membrane. However, despite the fact that ZY1-gp35 shows conservation with the central spike of close phages in some crucial amino acid sequences, the predicted 3D structures demonstrate notable variations in the host-binding module, which may explain why these genetically similar phages infect different types of hosts. In contrast to recognition and adsorption, endolysin-mediated cell wall disruption is a critical process in the late phase of the lytic cycle. As an N-acetylmuramidase, vB_BpuM-ZY1-encoded endolysin contains a highly conserved catalytic motif, which may ensure the efficiency of vB_BpuM-ZY1 in lysing the host. Overall, the sequence analyses of two genes critical for vB_BpuM-ZY1 during the early and late infection contribute to the understanding of the interactions between vB_BpuM-ZY1 and its host. However, several questions remain unanswered, such as (i) how vB_BpuM-ZY1 achieves host recognition when neither gene functional annotation nor phage morphological characterization revealed a tail fiber for host recognition, and (ii) how the endolysin of vB_BpuM-ZY1 crosses the host membrane to target peptidoglycan since no holin gene is identified in the vB_BpuM-ZY1 genome. Further investigations in the future are merited to address these questions.

## MATERIALS AND METHODS

### Host strain

The host strain, *B. pumilus*, was acquired from the Marine Culture Collection of China (MCCC, No.1A15722), which was isolated from mangrove sediments, in Fujian, China. *B. pumilu*s was cultured at 28°C with 200 rpm in Difco Marine Broth 2216 medium (Becton, Dickinson and Company, USA).

### Phage induction and purification

Mitomycin C was used to induce vB_BpuM-ZY1 from the *B. pumilus* strain. Briefly, bacterial strains were inoculated from plates into 15 mL tubes with liquid 2216 medium for activation and subsequently expanded in 200 mL liquid 2216 medium. After overnight incubation, the culture was added with mitomycin C at a final concentration of 0.5 µg/mL and then incubated for 2 h at 28°C. The suspension was centrifuged at 12,000 × *g* for 30 min at 4°C to remove cell debris. Thereafter, a final concentration of 10% (wt/vol) polyethylene glycol 8000 was added to the supernatant and kept at 4°C for more than 12 h. The phage precipitate was collected by centrifugation at 14,000 × g for 30 min at 4°C and resuspended in SM buffer (100 mM NaCl, 8 mM MgSO_4_, 50 mM Tris-HCl at pH 7.5), followed by filtering through a 0.22 µm filter.

### TEM observation

Phage morphology was observed using TEM. In brief, 10 µL of phage concentrate was dropped on a 200 mesh formaldehyde-coated copper electron microscope grid and adsorbed for 20 min. The phage was subsequently negatively stained with 1% (wt/vol) phosphotungstic acid for 1 min and air-dried for 2 h. The phage morphology was examined with a JEOL JEM2100F TEM (JEOL, Tokyo, Japan) at 80 kV. The plaque morphology was observed using the double-layer agar plate technique.

### Genomic sequencing and analysis

Phage nucleic acid extraction was performed according to the method proposed by Thurber et al. with some modifications (42). Briefly, the phage concentrate was treated with DNase-I (2.5 U/mL) for 1 h at room temperature, and then the reaction was terminated by incubating in a water bath at 65°C for 10 min. Subsequently, the phage particles were lysed by adding 1 vol of formamide for 30 min incubation at room temperature, and phage DNA was then extracted using the phenol-chloroform method. The phage DNA was fragmented to 300 bp using Covaris M220 (Covaris, USA), followed by library construction using NEBNext UltraTM DNA Library Prep Kit for Illumina (NEB, USA). The library was then sequenced using the Illumina Hiseq platform at Hanyu Bio-Tech (Shanghai, China). Sequencing reads were trimmed using Trimmomatic v0.36 to generate clean reads (43). The clean reads were then assembled using SOAPdenovo2 v2.04 and SPAdes v3.15.5 with default parameters, and the termini were identified by PhageTerm (44–46). The tRNAscan-SE v2.0 was used to predict tRNA genes (47). The open reading frames (ORFs) were predicted by Prodigal v2.60 and GeneMarkS v4.28 (48, 49). The functions of ORFs were subsequently annotated using blastp v2.2.30+ and EggNOG v5.0 (50, 51), and the genome map was visualized by Proksee (52).

### Protein-sharing network analysis

To cluster vB_BpuM-ZY1 with known viral populations, we performed a network analysis of vB_BpuM-ZY1 with other phages in the ProkaryoticViralRefSeq201-Merged database by vConTACT2 (v0.11.3) based on shared proteins. The network was visualized using Cytoscape (v3.9.1) (53, 54).

### Phylogenetic analysis

The TerL, conserved in *Caudovirales* (55), was used as a marker protein for phylogenetic analysis. 127 TerL sequences with different packaging mechanisms were included as reference sequences. These amino acid sequences were aligned using Muscle (v3.8.31) and trimmed by trimAL (v1.4.1), followed by the construction of phylogenetic trees by iqtree (v2.2.0) using the maximum-likelihood method with the bootstrap value of 1,000 (56–58). The phylogenetic tree was annotated by using the iTOL (v4) online tool (59).

To determine the taxonomic relationships, the genome sequences of vB_BpuM-ZY1 and 56 related phages (41 phages in the same cluster in the protein-sharing network and 15 other *Bacillus* phages) were analyzed using Virus Classification and Tree Building Online Resource (VICTOR) based on the GBDP method (60, 61). Classification at the family and genus level was performed using the OPTSIL program with the recommended clustering thresholds and an F-value (fraction of links required for cluster fusion) of 0.5 (60, 62). In addition, vB_BpuM-ZY1 genome-wide sequence similarity was analyzed by tBLASTx calculations using ViPTree, and a viral proteomic tree was constructed (63). For the construction of the proteomic tree, the dsDNA virus database (*n* = 5,632) with host-type Prokaryote was selected as the reference sequence, and the 60 viral sequences with top vB_BpuM-ZY1 genome similarity scores were selected for analysis.

The ANI between vB_BpuM-ZY1 and related phages was calculated, and the resulting ANI phylogenetic trees were created using OrthANI (v0.93.1) (64). Moreover, the intergenomic similarities were calculated using VIRIDIC, an algorithm that improves over the traditional BLASTN method (28). To detect synteny, a pairwise comparison between vB_BpuM-ZY1 and two closely related phage genomes obtained from the above analysis was performed using Clinker (v.0.0.23) (65).

### Sequence analysis and 3D structure modeling of central tail spike and endolysin

Identification and homology analysis of putative central tail spike protein (Baseplate assembly protein V) were performed using HHPred (https://toolkit.tuebingen.mpg.de/tools/hhpred) and InterPro/Pfam (https://www.ebi.ac.uk/interpro/) online tools (66, 67). Subsequently, the amino acid sequence of vB_BpuM-ZY1 central tail spike protein was aligned to the homologous central tail spike protein P2-gpV (AGG36531) and phi92-gp138 (CBY99567) by CLUSTALW (https://www.genome.jp/tools-bin/clustalw) and annotated using ESPript (https://espript.ibcp.fr/ESPript/cgi-bin/ESPript.cgi) (68, 69). The homologs of vB_BpuM-ZY1 endolysin were acquired by BlastP and were used to construct a phylogenetic tree along with vB_BpuM-ZY1 endolysin. The conserved structural domain of endolysin was detected by using NCBI CD-Search (70). The 3D structure of vB_BpuM-ZY1 central tail spike and endolysin were predicted using Alphafold2 (v2.3) (71, 72) and visualized by PyMOL (v2.5.4) (73).

## Data Availability

The genome sequence of vB_BpuM-ZY1 is deposited in the GenBank (accession number OR820920).

## SUPPLEMENTAL MATERIAL

Fig. S1 spectrum.00037-24-s0001.pdf Proteomic tree of vB_BpuM-ZY1 inferred by ViPTree.

Table S1 spectrum.00037-24-s0002.xlsx Functional annotation of vB_BpuM-ZY1 ORFs.

Table S2 spectrum.00037-24-s0003.xlsx Viral clusters of protein-sharing network.

ASM does not own the copyrights to Supplemental Material that may be linked to, or accessed through, an article. The authors have granted ASM a non-exclusive, world-wide license to publish the Supplemental Material files. Please contact the corresponding author directly for reuse.
