# Supplementary material for: Characterization and comparative genomic analysis of a marine Bacillus phage reveal a novel viral genus
Source: Microbiol Spectr. 2024 Aug 20;12(10):e00037-24. doi: 10.1128/spectrum.00037-24 (PMC11448403; doi:10.1128/spectrum.00037-24)
Supplement: Fig. S1 — Proteomic tree of vB_BpuM-ZY1 inferred by ViPTree. [file spectrum.00037-24-s0001.pdf]

Supplementary Figures

Characterization and Comparative Genomic Analysis of a Marine *Bacillus* Phage Reveal a Novel Viral Genus

Min Jin[1,3*], Meishun Yu[1], Xuejin Feng[1], Yinfang Li[1], Menghui Zhang[1,2*]

[1]State Key Laboratory Breeding Base of Marine Genetic Resource, Third Institute of Oceanography, Ministry of Natural Resources, Xiamen, China

[2] College of Ocean and Earth Sciences, Xiamen University, Xiamen, China

[3]Southern Marine Science and Engineering Guangdong Laboratory, Zhuhai, China

*Corresponding author (Menghui Zhang):

E-mail address: menghlike@163.com

*Corresponding author (Min Jin):

E-mail address: jinmin@tio.org.cn

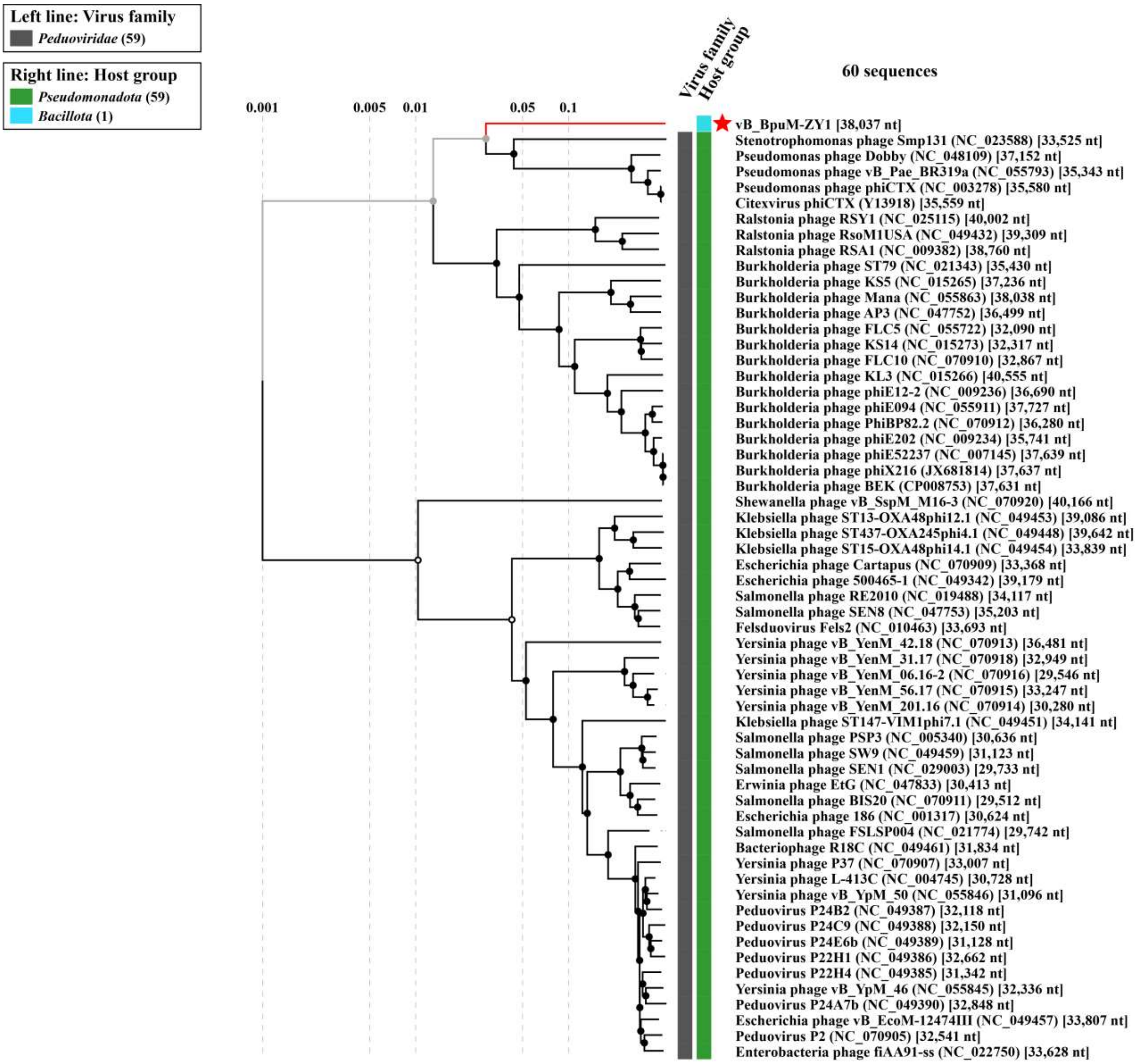

Fig. S1 Proteomic tree of vB_BpuM-ZY1 inferred by ViPTree.
